# Thermal Expansion and Magnetostriction Measurements at Cryogenic Temperature Using the Strain Gauge Method

**DOI:** 10.3389/fchem.2018.00072

**Published:** 2018-03-20

**Authors:** Wei Wang, Huiming Liu, Rongjin Huang, Yuqiang Zhao, Chuangjun Huang, Shibin Guo, Yi Shan, Laifeng Li

**Affiliations:** ^1^Institute of Advanced Metal Materials, School of Materials Science and Engineering, Tianjin University, Tianjin, China; ^2^State Key Laboratory of Technologies in Space Cryogenic Propellants, Technical Institute of Physics and Chemistry, Chinese Academy of Sciences, Beijing, China; ^3^Department of Science and Technology of Liquid Metal Material, School of Future Technology, University of Chinese Academy of Sciences, Beijing, China

**Keywords:** thermal expansion, magnetostriction, strain gauge method, cryogenic temperature, PPMS

## Abstract

Thermal expansion and magnetostriction, the strain responses of a material to temperature and a magnetic field, especially properties at low temperature, are extremely useful to study electronic and phononic properties, phase transitions, quantum criticality, and other interesting phenomena in cryogenic engineering and materials science. However, traditional dilatometers cannot provide magnetic field and ultra-low temperature (<77 K) environment easily. This paper describes the design and test results of thermal expansion and magnetostriction at cryogenic temperature using the strain gauge method based on a Physical Properties Measurements System (PPMS). The interfacing software and automation were developed using LabVIEW. The sample temperature range can be tuned continuously between 1.8 and 400 K. With this PPMS-aided measuring system, we can observe temperature and magnetic field dependence of the linear thermal expansion of different solid materials easily and accurately.

## Introduction

Cryogenic engineering has been extensively used in the fields of aerospace industry, superconducting technology, and large scientific instruments, etc. The physical performances of materials directly affect the safety and service time of the cryogenic system, among of which thermal expansion and magnetostriction, the strain responses of a material to temperature and a magnetic field, are the key thermodynamic properties closely related to specific heat and magnetization. Thus, accurate measurement of thermal expansion at low temperatures is necessary to ensure the security and reliability of the cryogenic systems. Also, these properties are very useful to investigate electronic and phononic properties, phase transitions, quantum criticality, and other interesting physical phenomena in cryogenic engineering and materials science (Ventura and Risegari, 2008; Inoue et al., 2014; Iwakami et al., 2014).

The approaches to estimate the thermal expansion of solid material can be classified, according to an extensometer, into quartz dilatometer, interferometer, optical lever, mechanical lever, magnetic meter, pointer meter, and capacitance micrometer methods, strain gauges, etc. (Clark, 1968; James et al., 2001; Kanagaraj and Pattanayak, 2003). The quartz dilatometer method is frequently used at low temperatures, because of its high accuracy (Corruccini and Gniewck, 1961; Deng and Xu, 2003; ASTM, 2006). However, it is expensive and complex to operate, and the sample size requirements are quite high (Tang et al., 2014). For a test specimen of large length, the accuracy may be affected by the potential temperature gradient along it, which then requires a cryostat to have high temperature uniformity.

The strain gauge method is also widely used to measure the thermal expansion of material at low temperatures (Liu et al., 2012, 2014). Compared with the quartz dilatometer method, the strain gauge method can measure multiple samples at the same time and has lower requirements of the test specimen size. However, traditional dilatometers cannot provide magnetic field environment easily and only been used in relative higher temperature range (>77 K) (Kaufman, 1963; Hannah and Reed, 1992).

Fortunately, Quantum Design Physical Properties Measurements System has been widely used for measurements of thermodynamic properties, such as specific heat, magnetization, electrical resistance, and thermal conductivity. In this report, we present the thermal expansion and magnetostriction measurements using the strain gauge method installed in a PPMS in the temperature range of 1.8–400 K and magnetic fields up to 14 T. The interfacing software and automation were developed using LabVIEW. Since the variation of the sensitivity coefficient of strain gauges at low temperatures is a key factor affecting the measurement accuracy, it was verified in the temperature range of 2–300 K by comparing the measured thermal expansion data of oxygen-free copper with the source data from National Institute of Standards and Technology (NIST), USA.

Also, using this PPMS-aided measuring system, we observed temperature and magnetic field dependence of the linear thermal expansion of NaZn_13_-type LaFe_13−x_Al_x_ compounds, which absolutely expands the practical application scope of this Multi-function testing system.

## Strain gauge method

It is well known that a strain gauge can measure the thermal strain on the surface of a test specimen to reflect the linear thermal expansion of material because it can transform the strain into the electric signal with a high precision. The strain caused by temperature fluctuation is defined as thermal output. Without a mechanical force imposed on the test specimen, the temperature change can bring about a length variation both in the test sample and in the strain gauge, which then cause the resistivity variation of the strain gauge correspondingly. And the strain variation induced by the net temperature can be expressed as follows (Poore and Kesterson, 1978; Walsh, 1997; Micro-Measurements, 2007):

(1) $$\begin{array}{r} \varepsilon =\frac{\beta_{\text{g}}}{F_{\text{g}}}\Delta T+\left(\alpha_{\text{s}}-\alpha_{\text{g}}\right)\;\Delta T \end{array}$$

where ε is the apparent strain of strain gauge, β_*g*_ is the thermal coefficient of resistivity of the grid material, *F*_*g*_ is the gauge factor of strain gauge, α_*s*_ − α_*g*_ is the difference in coefficient of thermal expansion (CTE) between the tested sample and gauge, respectively, and Δ*T* is the temperature change from arbitrary initial reference temperature. For two same types of strain gauges installed on test sample (α_*s*_) and reference sample (α_*r*_), respectively, the apparent strains can be expressed as:

(2a) $$\begin{array}{r} \varepsilon_{\text{s}}=\frac{\beta_{\text{g}}}{F_{\text{g}}}\Delta T+\left(\alpha_{\text{s}}-\alpha_{\text{g}}\right)\;\Delta T \end{array}$$

(2b) $$\begin{array}{r} \varepsilon_{\text{r}}=\frac{\beta_{\text{g}}}{F_{\text{g}}}\Delta T+\left(\alpha_{\text{r}}-\alpha_{\text{g}}\right)\;\Delta T \end{array}$$

Subtracting Equation (2b) from (2a), and rearranging:

(3) $$\begin{array}{r} \alpha_{s}=\frac{\varepsilon_{S}-\varepsilon_{r}}{\Delta T}+\alpha_{r} \end{array}$$

Thus, the CTE of test sample can be obtained with the accurately measured $\varepsilon_{S}-\varepsilon_{r}$ and Δ*T*, as well as the known CTE of reference sample α_*r*_. In this paper, we use fused silica of high purity (>99.99%) as the reference sample in the measurement of thermal expansion due to its very low and almost temperature-independent mean linear CTE (5.5 × 10 ^−7^/K) (Kirby and Hahn, 1971).

## Experimental procedures and methods

**Figure 1:**
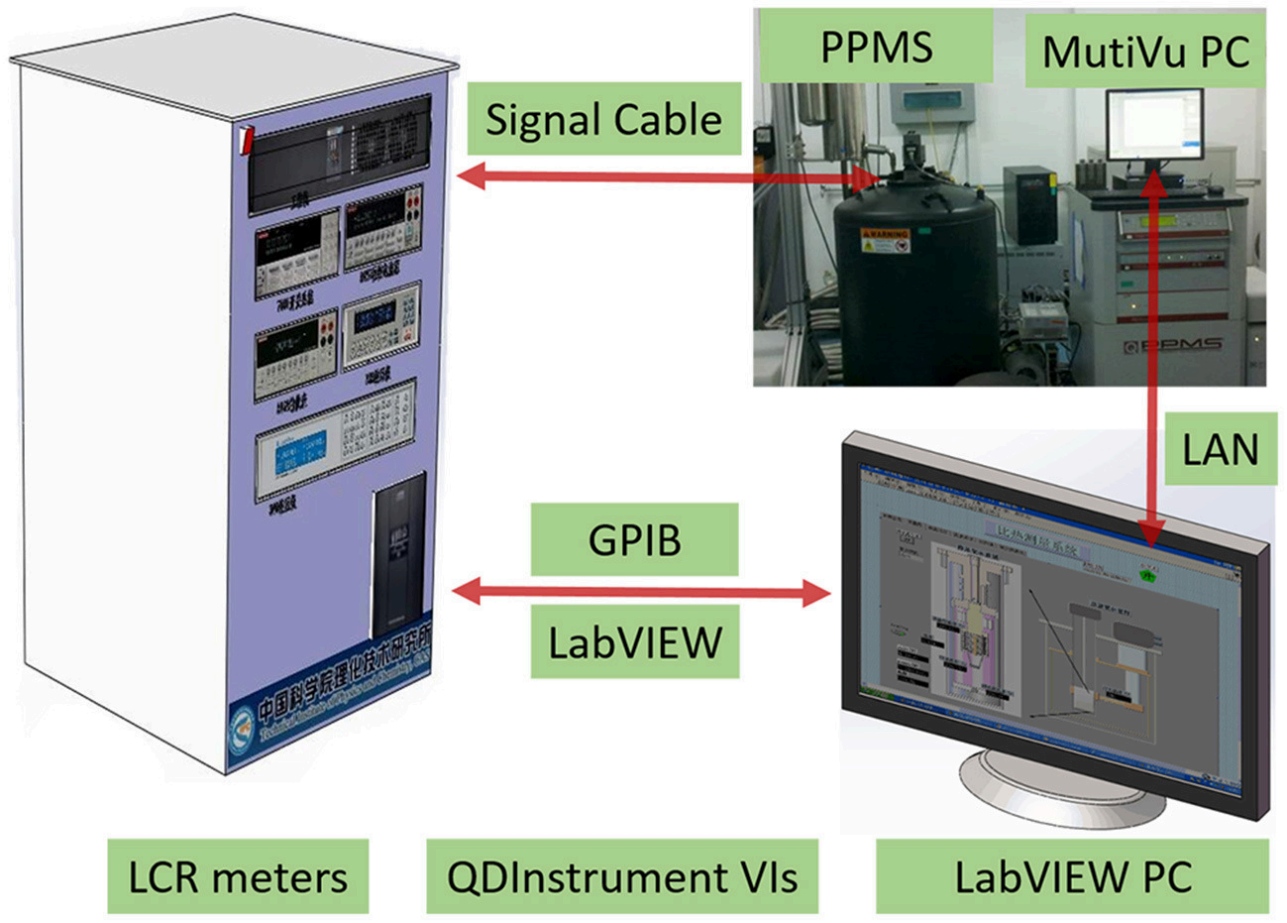
Schematic of measurement system for thermal expansion of solid materials.

The schematic view of the measurement system is shown in Figure 1, which consists of a PPMS, nanovoltmeters, and a LabVIEW PC.

With the help of LabVIEW, we can design experiments using our own measurement electronics when working on the general purpose platforms of the PPMS (Quantum Design Inc., 2002, 2013). Firstly, nanovoltmeters (KEITHLEY 2182A, USA) can monitor the electrical signals of samples in PPMS via a GPIB interface simultaneously. And then a LabVIEW computer attached to nanovoltmeters also via a GPIB interface. The QDInstrument VIs and QDInstrument.dll are installed on this computer and communicate over .NET. In brief, the QDInstrument program is a translator with a .NET interface for communication with the LabVIEW VI and an OLE interface for communication with MultiVu. The DLL is configured to address the QDInstrument_server.exe program running on the MultiVu computer. This is done via connecting an Ethernet cable directly between the two computers in the absence of a LAN. The server program in turn communicates over OLE with the MultiVu application. Finally, by controlling both external devices as well as PPMS's state (temperature and magnetic field) on a separate LabVIEW computer from MultiVu, we can observe the temperature and magnetic field dependence of the linear thermal expansion of different solid materials easily and accurately.

**Figure 2:**
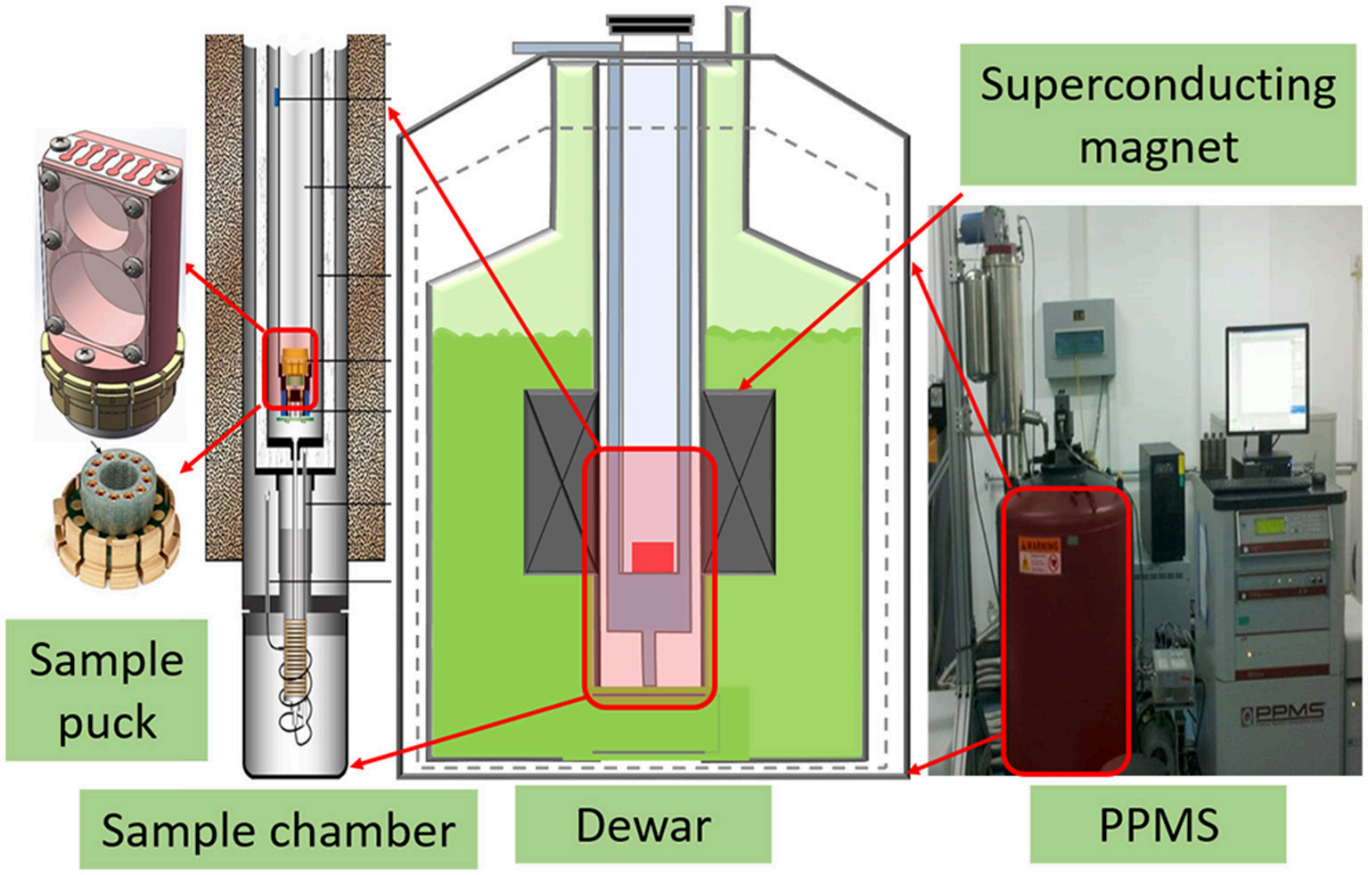
Schematic of the sample chamber.

The sample puck, as demonstrated in Figure 2, is redesigned to meet the special requirement of thermal expansion measurement. The whole sample puck is emerged into sample chamber of PPMS to cool the test sample. The temperature and magnetic field can be adjusted by the PPMS in the range of 1.8–400 K and ±14 T. The dimensions of the test sample can be tuned from 3 × 3 × 3 to 8 × 8 × 8 mm. Its relatively small dimensions can reduce the difficulty in machining and the influence of the non-uniform temperature field on the measurement accuracy. The reference sample with the same dimensions is made from fused silica of 99.99% purity. To measure the thermal expansion, test sample and reference sample are placed on the sample puck stress-free. Two identical strain gauges are bonded on the center of the test sample surface and the reference sample surface, respectively, using a high performance adhesive M-Bond 610. This adhesive is capable of forming thin, hard gluelines for maximum fidelity in transferring strains from the sample surface to the gauge.

The Ni-Cr resistive elements BB120-2AA250 (11) Karma foil strain gauges (Zhonghang Electronic Measuring Instruments Co. Ltd., China) were used. The nominal resistance is (119.9 ± 0.1) Ω, and the nominal sensitivity coefficient is 1.74 ± 0.01. When the measurement starts, the automated program controls the temperatures and magnetic field of the PPMS at the different rates. And the thermal output of the strain gauges and the temperature are recorded by nanovoltmeters. Then the LabVIEW computer can save the temperature and magnetic field dependence of the linear thermal expansion of tested sample.

## Results and discussion

**Figure 3:**
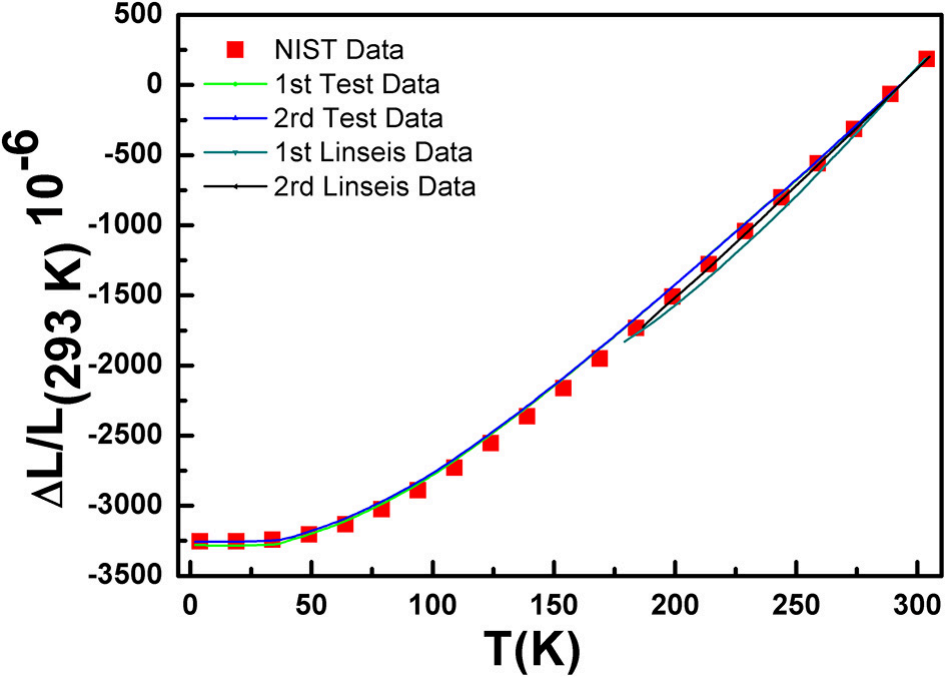
Experimental and published values of thermal expansion of copper.

The accuracy of the CTE measurement mainly depends on the accuracy of the strain gauges, the nanovoltmeters and the procedure of the gauges installation. To verify the accuracy of this test apparatus and method, the thermal expansion of oxygen-free copper are measured in the temperature range of 2–300 K on this apparatus and quartz dilatometer (Linseis L75), respectively. Also, the Comparisons between experimental and published values of thermal expansion of copper are displayed in Figure 3 (Kirby and Hahn, 1969). The results of thermal expansion of copper matched closely with reference data, and the maximum error for instantaneous coefficients is 1.1 × 10^−6^ K^−1^, which is smaller than that testing using quartz dilatometer. At the same time, to validate the reliability of the measurement, experiments are repeated from 2 – 300 K. We can see that there is no significant difference of the thermal expansions (<2%) between the two tests, which shows that the whole measurement system is applicable in the temperature range of 2–300 K. Also, this method is available for other special structure materials such as low symmetry samples and orientation preferred samples with relatively smooth and nonporous surfaces.

In the last several years, due to the giant magnetocaloric effect (MCE) and the intriguing negative thermal expansion (NTE), NaZn_13_-type compounds LaFe_13−x_Al_x_ have aroused extensive attention (Fujieda et al., 2002; Shen et al., 2009; Li et al., 2014; Wang et al., 2015). The MCE of this materials is chiefly due to the itinerant-electron metamagnetic (IEM) transition, i.e., a magnetic-field-induced phase transition from the paramagnetic state to the ferromagnetic state. The NTE effect is a thermo-physical property and attributed to the magnetovolume effect (MVE). Nevertheless, very few report explores the relationship between the change of volume expansion and external magnetic field, i.e., magnetostriction, in LaFe_13−x_Al_x_ compounds.

**Figure 4:**
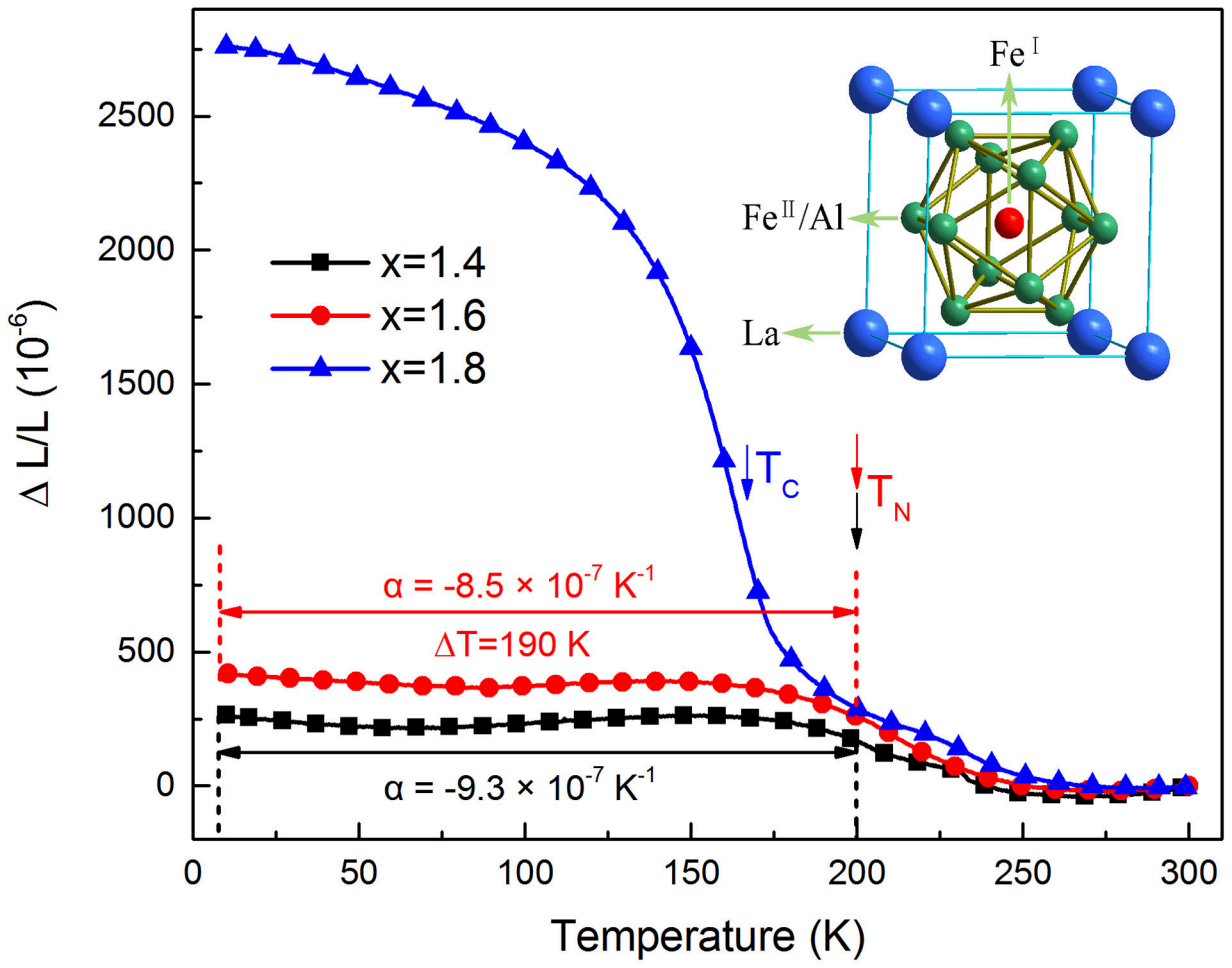
Temperature dependence of linear thermal expansions ΔL/L (reference temperature: 300K) of LaFe_13−x_Al_x_.

Generally, the large magnetostriction originates from the interaction between atom magnetic moments. LaFe_13−x_Al_x_ compounds (1.04 < x < 7.02) share the same NaZn_13_-type crystalline structure while showing different magnetic states (antiferromagnetic state, ferromagnetic state and paramagnetic state). Specificly, LaFe_13−x_Al_x_ (1.0 < x < 1.8) show an antiferromagnetic state at low temperature. And the antiferromagnetic ordering is very vulnerable and can be turned into the ferromagnetic state easily when bringing an external magnetic field. Different magnetic states have different magnetic moments. So a large magnetostriction LaFe_13−x_Al_x_ of compounds is caused by these magnetic phase transition. In order to better understanding the magnetostrictive property, as displayed in Figure 4, the linear thermal expansion data (ΔL/L 300 K) of LaFe_13−x_Al_x_ (x = 1.4, 1.6, and 1.8) compounds were measured using this PPMS-aided measuring system over a temperature range of 10–300 K firstly (Zhao et al., 2017). For LaFe_13−x_Al_x_ (x = 1.4 and 1.6) below the Neel temperature, it shows that the CTE is very constant across a wide temperature range (10–200K). However, for the sample LaFe_11.2_Al_1.8_, ΔL/L rises with decreasing temperature in the whole temperature range examined. Specifically, ΔL/L grows gradually around room temperature, then increases rapidly, and again grows slowly with decreasing temperature, indicating that NTE occurs in the whole range measured (2–300 K). It is worth noting that the ΔL/L vs. T curves are different from one another, which demonstrates that the NTE properties are affected by the partial substitution of Fe by Al sensitively.

**Figure 5:**
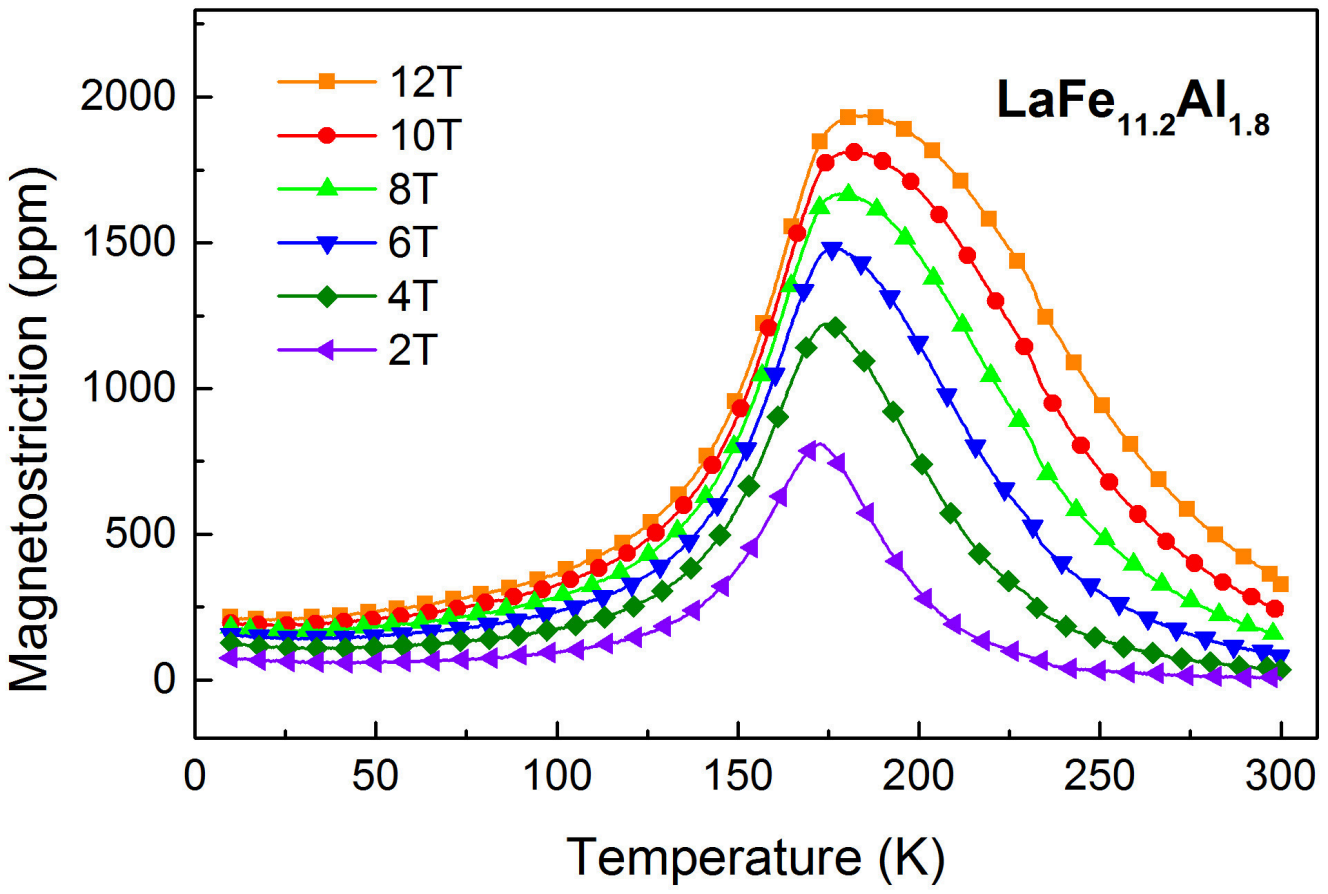
Temperature dependence of the magnetostriction λ(T) in LaFe_11.2_Al_1.8_ at different magnetic fields.

Also it is worth mentioning that the linear magnetostriction of this material is isotropic. Because the magnetostriction measurements have been conducted along the parallel and perpendicular directions to the magnetic field separately and the anisotropic magnetostriction is negligible. Figure 5 gives the temperature dependence of the linear magnetostriction λ(T) of LaFe_11.8_Al_1.8_ compounds between 10 and 300 K. It is noted that all the magnetostrictions of the sample in different magnetic fields exhibit a sudden increase with the decrease of the temperature and then, decrease rapidly with following temperature decline. In addition to this, it is apparent that the temperature with giant magnetostriction shifts to higher temperature as the magnetic field increases. Importantly, giant linear magnetostriction values higher than 1,000 ppm are observed when the magnetic field exceeds 4T in a relatively wide temperature range. Especially, it can reach up to 1,900ppm at 12T. Interestingly, the peak value of magnetostriction of the LaFe_11.2_Al_1.8_ compound is larger than the maximum of the typical magnetostriction material, Terfenol-D alloy (Grössinger et al., 2014).

**Figure 6:**
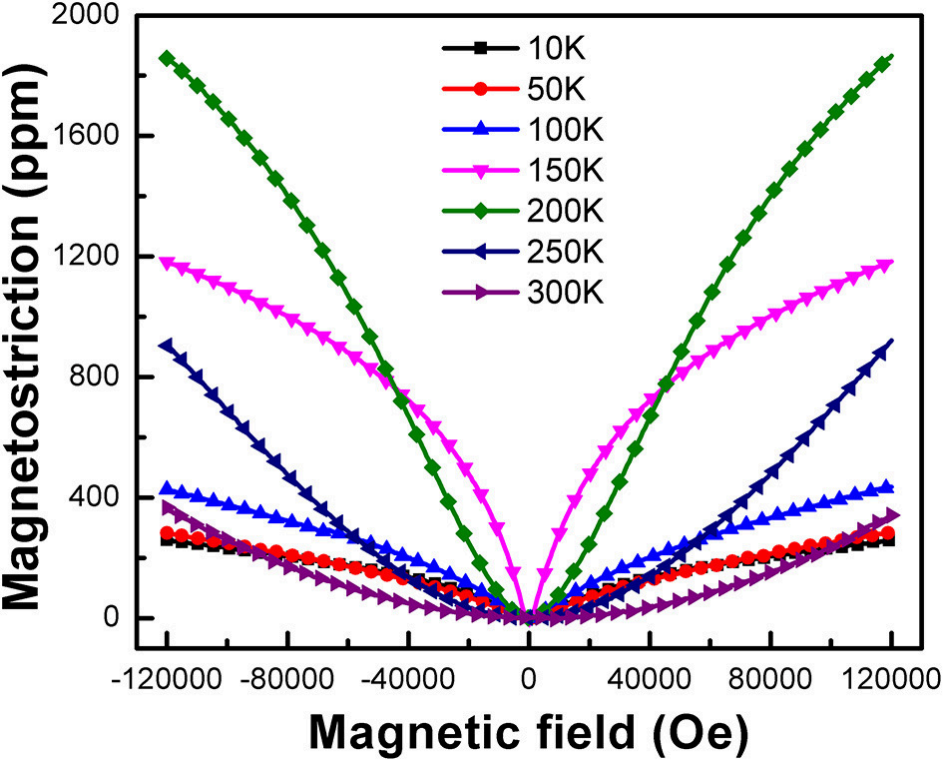
Magnetic field dependence of the magnetostriction λ(H) of LaFe_11.2_Al_1.8_ compounds at different temperatures.

To further explore the magnetostrictive property, the magnetic field dependence of the magnetostriction λ(H) under different temperatures for LaFe_11.2_Al_1.8_ sample have been investigated in Figure 6. Apparently, with the increase of the magnetic field, the λ(H) raises gently at 300 K and reaches to 340 ppm under 12T finally, suggesting the magnetostrictive effect of this material is extremely weak at room temperature. But when the temperature decreases, the λ(H) increases rapidly with increasing magnetic field. And when the temperature comes up to 200 K, the λ(H) is almost increase linearly with the increase of magnetic field. Besides, when the temperature falls below 150K, it is observed that below 4T, the magnetostriction increases quickly with the increase of the magnetic field. However, when magnetic field exceeds 4T, the magnetostriction increases slowly and becomes lower than that in 200 K. And after that, the magnetostriction is rapidly decreased with temperature decline from 100 to 10 K. The results indicate that the magnetostriction of this sample is very sensitive and the giant magnetostriction happens in a relatively narrow temperature range.

## Conclusion

In summary, an experimental setup with a PPMS based on the strain gauge method has been developed to measure the thermal expansion and magnetostriction of solid materials at low temperatures (1.8–400 K). The relatively small dimensions of the test sample can save the liquid helium and reduce the impact of the non-uniform temperature field on measurement accuracy. The measurement accuracy was verified in the temperature range of 2–300 K by comparing the measured thermal expansion data of oxygen-free copper with the source data from NIST. Also, using this PPMS-aided measuring system, we observed temperature and magnetic field dependence of the linear thermal expansion of NaZn_13_-type LaFe_13−x_Al_x_ compounds. Such Multi-function testing system with a broad measurement temperature range will open up an intriguing research frontier for exploring potential magnetostrictive and NTE materials.

## Author contributions

WW and HL: Designed experiments; YZ, SG, and YS: Carried out experiments; HL and CH: Analyzed experimental results; WW, RH, and LL: Wrote the manuscript.

### Conflict of interest statement

The authors declare that the research was conducted in the absence of any commercial or financial relationships that could be construed as a potential conflict of interest. The reviewer KL and handling Editor declared their shared affiliation.
